# No Varicella Zoster Virus Infection among Mpox Cases in Antwerp, Belgium

**DOI:** 10.4269/ajtmh.23-0353

**Published:** 2023-10-16

**Authors:** Jasmine Coppens, Laurens Liesenborghs, Koen Vercauteren, Marjan Van Esbroeck, Christophe Van Dijck

**Affiliations:** Department of Clinical Sciences, Institute of Tropical Medicine, Antwerp, Belgium

## Abstract

Several studies in tropical settings have reported that ∼20% of patients infected with Monkeypox virus (MPXV) also tested polymerase chain reaction (PCR) positive for Varicella zoster virus (VZV). Researchers have hypothesized that VZV infection predisposes to monkeypox (mpox), or vice versa, or that MPXV triggers the reactivation of VZV. We tested samples for VZV from a cohort of patients infected with clade IIb MPXV diagnosed between May 23 and October 14, 2022 in Antwerp, Belgium. Leftover DNA extracts of skin lesion samples from 108 mpox patients were tested with in-house PCR for VZV. No VZV infections were found. The absence of concurrent VZV-MPXV infections in our cohort indicates that VZV did not cocirculate in the population at risk for MPXV during the Belgian 2022 outbreak, but also that MPXV does not commonly trigger reactivation of latent VZV in adult men.

Monkeypox (mpox) is an endemic zoonosis caused by Monkeypox virus (MPXV) clade I in Central Africa and clade II in West Africa.^1^ In these settings, MPXV primarily affects children and young adults.^1^ In 2022, MPXV subclade IIb caused a worldwide outbreak of sexually associated mpox among men who have sex with men.^1^ Monkeypox can be difficult to distinguish clinically from infections caused by Varicella zoster virus (VZV) because of their similar presentation of skin rash with or without fever.^2^ Both infections present with vesicular skin lesions that can be itchy and painful. The rash caused by VZV can be either generalized after primary infection (varicella or chickenpox), or localized to one or more dermatomes upon viral reactivation (herpes zoster or shingles). Likewise, the rash caused by MPXV can be either generalized or more localized to a single body region, depending on the disease severity and the viral clade causing the infection. Clade I MPXV has a tendency to cause a more generalized mpox rash compared with clade II MPXV.^1^ In addition, mpox vesicles do not always evolve into the typical umbilicated pustules that distinguish them from varicella vesicles. Furthermore, mpox epidemics in endemic countries often occur in populations in which outbreaks of chickenpox are common, with considerable overlap in the age of affected populations.^1^^–^^4^ Therefore, when laboratory testing for VZV and MPXV is not available, it can be challenging to differentiate between the two infections based solely on clinical symptoms.^2^

Interestingly, in certain situations when testing was performed, concurrent infections of VZV and MPXV were reported.^5^^–^^7^ Two reports from the Democratic Republic of the Congo found 151 of 782 patients with mpox (19.3%) and 134 of 534 patients with mpox (25.1%) with a concurrent VZV infection.^5^^,^^6^ Similarly, 9 of 33 patients with mpox (27.3%) in an outbreak in Nigeria also had VZV detected in samples of their skin lesions.^7^ More recently, 5 of 25 patients (20%) infected with clade IIb MPXV during the 2022 outbreak in Brazil also tested positive for VZV.^8^ The reasons for this remarkably high occurrence of combined infections are unclear. Researchers have hypothesized that chickenpox is a risk factor for mpox, or vice versa.^4^^,^^9^ An alternative explanation could be that MPXV provokes reactivation of latent VZV (shingles). Diagnoses of VZV–MPXV coinfections may have been missed during the 2022 mpox outbreak, because testing was primarily targeting MPXV in the population at risk for mpox.

Here, we evaluate the presence of VZV in skin lesion samples from patients infected with MPXV clade IIb in a sexual health clinic in Antwerp, Belgium. Of 179 patients diagnosed with mpox between May 23 and October 14, 2022, 162 (90.5%) consented to the use of their data and samples for research. One hundred fifty-one patients (93.2%) had skin lesions at the time of diagnosis. For 108 of these 151 patients (71.5%), leftover DNA extracts were available of skin lesion samples with MPXV polymerase chain reaction (PCR) cycle threshold (ct) values < 34. These 108 DNA extracts were analyzed with real-time PCR targeting the VZV *orf38* gene.^10^ Samples with weak positive VZV PCR results (ct value ≥ 38) were retested and considered negative if the second PCR result was negative.

The median age of the 108 included mpox patients was 38.5 years (range, 21–66 years, Table 1). The majority (87%) had less than 25 skin lesions. One patient was immunocompromised as a result of HIV, with a CD4 count of 195 cells/mm^3^. The MPXV PCR ct values of the included skin lesion samples ranged between 14.90 and 33.97, with a median of 20.82. All samples tested negative for VZV.

**Table 1:** Characteristics of patients and samples included in this study

Characteristic	Overall (*N* = 108)
Age, years	
Median [range]	38.5 [21.0–66.0]
Missing, *n* (%)	2 (1.9)
Male gender, *n* (%)	108 (100)
Sexual preference, *n* (%)	
Homosexual	98 (90.7)
Heterosexual	7 (6.5)
Bisexual	3 (2.8)
HIV status	
Unknown, *n* (%)	7 (6.5)
HIV negative, *n* (%)	62 (57.4)
HIV positive, *n* (%)	39 (36.1)
CD4 < 200 cells/mm^3^, *n*/*N* (%)	1/39 (2.6)
CD4 ≥ 200 cells/mm^3^, *n*/*N* (%)	32/39 (82.1)
CD4 unknown, *n*/*N* (%)	6/39 (15.4)
Skin lesions present, *n* (%)	108 (100)
Duration of skin lesions prior to clinic attendance	
Median no. of days [range]	4.00 [1.00–21.0]
Missing, *n* (%)	3 (2.8)
Lesion type, *n* (%)	
Macule	14 (13.0)
Papule	32 (29.6)
Vesicle	31 (28.7)
Pustule	50 (46.3)
Crust	56 (51.9)
Ulcer	61 (56.5)
Lesion location, *n* (%)	
Face	34 (31.5)
Lips	10 (9.3)
Oral cavity	7 (6.5)
Chest	22 (20.4)
Abdomen	16 (14.8)
Back	28 (25.9)
Arms	31 (28.7)
Hand palms	19 (17.6)
Buttocks	17 (15.7)
Upper legs	22 (20.4)
Lower legs	10 (9.3)
Soles of feet	5 (4.6)
Perianal	35 (32.4)
Pubis	25 (23.1)
Scrotum	29 (26.9)
Penis	46 (42.6)
No. of lesions estimated by clinician, *n* (%)	
0–4	35 (32.4)
5–25	59 (54.6)
26–100	12 (11.1)
> 100	1 (0.9)
Missing	1 (0.9)
MPXV PCR positive, *n* (%)	108 (100)
Median MPXV PCR ct value [range]	20.82 [14.90–33.97]
VZV PCR positive	0 (0)

ct = cycle threshold; MPXV = Monkeypox virus; PCR = polymerase chain reaction; VZV = Varicella zoster virus.

In contrast to the reports from the Democratic Republic of the Congo,^5^^,^^6^ Nigeria,^7^ and Brazil,^8^ our study did not find coinfections of VZV and MPXV among Belgian mpox patients. The reasons for these discrepancies remain speculative. The absence of coinfections might be attributed to differences in the age profile and the epidemiology of both viruses in the different regions. In Belgium, as in many other temperate countries without routine VZV vaccination, VZV is a common childhood infection, causing chickenpox in almost all children by the age of 5 years.^11^ The 2022 mpox epidemic affected adult men, most of whom can be assumed to have developed immunity against VZV.^12^ Therefore, adult VZV primary infections are rare. This is in contrast to more tropical regions, where VZV primary infections often occur at a later age.^3^ Our data provide relative evidence against the hypothesis that MPXV triggers reactivation of latent VZV, leading to shingles. If MPXV were indeed responsible for reactivating VZV in ∼20% of cases, it would be highly unlikely not to observe any MPXV–VZV coinfection in our cohort. Our findings may not be generalizable to settings with a greater prevalence of immunodepression resulting from uncontrolled HIV, malnutrition, or other causes, as these conditions may increase the risk for coinfection upon exposure to VZV and MPXV, or reactivation of VZV. In addition, we do not know whether our findings hold true for clade I MPXV, because we only studied clade IIb–infected individuals. However, under the conditions observed in a temperate, high-income country, we conclude that clade IIb MPXV infection does not commonly provoke the reactivation of latent VZV in immunocompetent individuals.
